# Direct Patterned Zinc-Tin-Oxide for Solution-Processed Thin-Film Transistors and Complementary Inverter through Electrohydrodynamic Jet Printing

**DOI:** 10.3390/nano10071304

**Published:** 2020-07-03

**Authors:** Heqing Ye, Hyeok-Jin Kwon, Xiaowu Tang, Dong Yun Lee, Sooji Nam, Se Hyun Kim

**Affiliations:** 1 School of Chemical Engineering, Yeungnam University, Gyeongsan 38541, Korea; yeheqing5420@gmail.com (H.Y.); tangxiaowu@naver.com (X.T.); 2 Department of Chemical Engineering, Pohang University of Science and Technology, Pohang 37673, Korea; hj1370@postech.ac.kr; 3 Department of Polymer Science and Engineering, Kyungpook National University, Daegu 41566, Korea; 4 Flexible Electronics Research Section, Electronics and Telecommunications Research Institute, Daejeon 34129, Korea; 5 Advanced Device Technology, University of Science & Technology, Electronics and Telecommunications Research Institute, Daejeon 34129, Korea

**Keywords:** zinc-tin-oxide, electrohyrodynamic jet printing, oxide thin-film transistors, complementary inverter

## Abstract

The solution-processed deposition of metal-oxide semiconducting materials enables the fabrication of large-area and low-cost electronic devices by using printing technologies. Additionally, the simple patterning process of these types of materials become an important issue, as it can simplify the cost and process of fabricating electronics such as thin-film transistors (TFTs). In this study, using the electrohydrodynamic (EHD) jet printing technique, we fabricated directly patterned zinc-tin-oxide (ZTO) semiconductors as the active layers of TFTs. The straight lines of ZTO semiconductors were successfully drawn using a highly soluble and homogeneous solution that comprises zinc acrylate and tin-chloride precursors. Besides, we found the optimum condition for the fabrication of ZTO oxide layers by analyzing the thermal effect in processing. Using the optimized condition, the resulting devices exhibited satisfactory TFT characteristics with conventional electrodes and conducting materials. Furthermore, these metal-oxide TFTs were successfully applied to complementary inverter with conventional p-type organic semiconductor-based TFT, showing high quality of voltage transfer characteristics. Thus, these printed ZTO TFT results demonstrated that solution processable metal-oxide transistors are promising for the realization of a more sustainable and printable next-generation industrial technology.

## 1. Introduction

Thin-film transistors (TFTs) are regarded as the key components in flat-panel display (FPD) applications, such as active matrix liquid crystal displays and active matrix organic light-emitting diode displays [1]. In recent days, the development of TFT has become an important part of FPDs with high resolution and large size, thereby pushing the traditional amorphous Si (a-Si) TFTs to their limit [2,3]. Accordingly, metal oxide-based TFTs might be now one of the most promising technologies in the next-generation display technologies because of their high carrier mobility, excellent transparency, satisfactory uniformity, and reasonable electrical reliability/stability [4,5,6]. Compared with traditional covalent semiconductor- (e.g., a-Si) based TFTs, whose carrier transport paths comprise sp^3^ orbitals with strong directivity, metal oxide TFTs form a charge-transfer path from the metal orbitals to the oxygen orbitals with a strong degree of iconicity [7]. Therefore, the conduction band minimum is actually formed because of highly dispersive unoccupied metal orbitals, whereas the valence band maximum is composed because of fully occupied and localized oxygen orbitals [5,8]. Those vacant metal orbitals are spherical (i.e., non-directional) and exhibit large spatial spread, inducing high electron-transport characteristics in the TFT operation [9]. 

However, despite the aforementioned high-performance characteristics, most high-performance metal oxide-based TFTs used in well-established industries thus far are manufactured using conventional vacuum deposition processes. These types of manufacturing processes limit their potential applications in large-area electronics because of the high-cost of equipment and small deposition area in processing. Additionally, complex processes (e.g., photolithography and shadow mask) are also needed to produce the patterns that are required for each device application. However, the solution process method that utilizes a metal oxide precursor offers many advantages such as large-area fabrication, equipment and process step simplicity, roll-to-roll capability, atmospheric processing, and low cost [8,10]. Particularly, direct pattering process with printing technology reduces the time and cost of the process, thereby circumventing the vacuum and photolithography process. In addition, it is an environmentally friendly technology that can help reduce chemical wastes [4]. 

Compared with the conventional ink-jet printing technique, the electrohydrodynamic (EHD) jet printing process can provide high-resolution micro- to nanoscale patterns using well-modified droplets [11,12,13]. The meniscus of the droplet that hangs on the nozzle tip can be deformed because of the applied electrostatic field, thereby elongating the ink to generate a discrete droplet or jet on the substrate. According to the electrostatic-field intensity and ink properties, various jetting modes, which are capable to directly write materials such as organic, metallic, and metal-oxide materials, are developed [14,15,16,17]. Especially, the stable cone-jet mode of the EHD jet printing reduces the diameter of droplets to smaller than that of the nozzle tip to fabricate patterns on the target substrate. 

In this study, we examined solution-processed zinc-tin-oxide (ZTO) semiconductors as the active layers of metal oxide TFT, with different annealing conditions. In addition, we used the EHD-printed ZTO as the active layers of TFT with various source(S)/drain(D) electrode materials and also applied the complementary inverter with organic semiconductor-based TFTs. ZTO precursors for printing were synthesized via mixing with zinc acrylate and tin chloride. Conventional metallic material, Al, and previously reported printable multi-walled carbon nanotubes (MWCNT)/Triton X-100 composites were utilized for fabricating S/D electrodes [18,19]. The processing and printing parameters, such as the annealing condition, flow rate, voltage, and printing speed, were investigated for the optimal formation of patterned ZTO active layers. Consequently, the optimized EHD jet-printed ZTO showed low-hysteresis driving behaviors with the field-effect mobility (*μ_FET_*) of 1.35 ± 0.14, 0.52 ± 0.08 cm^2^/V s for Al- and MWCNT-based devices. Moreover, these well-operated oxide-based TFT devices (mainly electrons were transported) were successfully applied to the fabrication of complementary inverters by combining with organic material-based transistors (holes were mainly transported). Consequently, we believe that this research will contribute not only to the production of EHD printing-based oxide semiconductors for TFTs, but also to the fabrication of electronic devices based on the properties of oxide- and organic-based materials.

## 2. Materials and Methods 

### 2.1. Materials and Sample Preparation

Zinc acetate dehydrate, tin chloride, and 2-methoxyethanol were purchased from Sigma-Aldrich (St. Louis, MO, USA) and used as received without further purification. The solution-processable ZTO semiconductor precursor was synthesized using totally 0.4 M of zinc acetate dehydrate and tin chloride in the mole ratio of 1:1, in 2-methoxyethanol. Before depositing this solution, we stirred it to achieve a homogeneous-solution state. The MWCNT-based solution was synthesized following a method in a previously conducted study [19]. Briefly, the MWCNTs (diameter: 10–20 nm, length: 5–20 µm, Nanolab from Newton MA, USA) and Triton X-100 (polyethylene glycol tert-octylphenylether, TX100, Sigma-Aldrich, St. Louis, MO, USA) were dispersed in ethanol (Sigma-Aldrich, St. Louis, MO, USA) to prepare MWCNTs’ electrode ink. Heavily doped n-type Si wafers (resistivity < 0.005 Ω cm, Namkang hi-tech, Seongnam-si, Korea) were used as both gate electrodes and substrate for device fabrication. The wafers were rinsed using isopropyl alcohol (IPA) and cleaned using a UV-ozone cleaner for 20 min. Subsequently, the gate dielectric layers were deposited through the atomic layer deposition (ALD)-processed 50-nm alumina (Al_2_O_3_) using a previously reported method [20,21]. The gate dielectric-deposited wafer was then clipped to the size of 2 × 2 cm^2^. These clipped samples were cleaned using boiled acetone for 10 min. Subsequently, the samples were ultra-sonicated in acetone and IPA for 20 min, dried under a nitrogen stream, and further cleaned via UV-ozone exposure for 15 min.

### 2.2. Fabrication of ZTO Patterning and Solution-Processed Oxide TFTs

ZTO patterns were produced by directly writing, using the previously created ZTO precursor solution via the EHD jet printing process. The ZTO precursor ink was taken in a glass syringe with the nozzle diameter of 200 μm and loaded in the EHD jet printing machine (Enjet, Gyeonggi-do, Korea). Notably, the flow rate and working distance in this study were optimized at 0.15 μL/min and 280 μm, respectively. A power supply was used to apply an electric field between the nozzle and substrate, which served as the ground. The EHD printing was performed in various jetting modes by adjusting direct current (DC) voltage values and at various printing speeds between 5 and 50 mm/s in ambient air conditions. Subsequently, the active layers were annealed at various thermal conditions (300°C, 400 °C, and 500 °C) to yield metal-oxide active layers. The Al or MWCNTs’ electrodes were printed for using the source (S) and drain (D) electrodes in the same manner as in a previously reported condition [19].

### 2.3. Fabrication of Complementary Inverters

Heavily n-doped Si wafers (resistivity < 0.005 Ω· cm) with thermally grown 300 nm SiO_2_ were utilized as the substrates for complementary inverters, and Si and SiO_2_ were utilized as input voltage (*V_in_*) electrode and gate dielectrics, respectively. Before fabrication of complementary inverters, the Si wafers were cleaned with sequential boiling with acetone for 10 min. Then, the boiled wafers were further cleaned with ultra-sonicator with acetone and IPA for 20 min and dried under a nitrogen stream. Before printing the ZTO layers, the substrates further cleaned via UV-ozone exposure for 15 min. For fabrication of complementary inverters, ZTO patterns were firstly printed through the EHD jet printing process on the active layer region of n-type transistor on n-doped Si with thermally grown 300 nm SiO_2_. Then, the 2,9-di-decyl-dinaphtho-[2,3-b:2′,3′-f]-thieno-[3,2-b]-thiophene (C_10_-DNTT), which was synthesized with the previously reported method [22], was deposited on the p-type transistor’s active layer region as 50 nm thick using the organic molecular beam deposition (deposition rate = 0.1 Å s^−1^, vacuum pressure = 10^−6^ Torr, substrate temperature = 25 °C). The 50-nm-thick Au electrodes for contact of supply voltages (*V_DD_*), ground, and output voltage (*V_out_*) were deposited through the thermal evaporation (deposition rate = 2 Å·s^−1^, vacuum pressure = 10^−6^ torr, substrate temperature = 25 °C) on the active deposited substrates. 

### 2.4. Characterization

The meniscus of EHD jet printing was monitored using a charge-coupled device camera of EHD printer. The printed ZTO patterns height properties were analyzed by the alpha-step (NanoMap-PS) and optical microscope (OM, Nikon ECLIPSE LV100ND, Tokyo, Japan). The morphologies of the deposited ZTO were analyzed using OM and scanning electron microscope (SEM, Hitachi S4800, Ibaraki, Japan). The ZTO film was characterized via X-ray diffraction (XRD, X’pert Pro MPD, Panalytical, Malvern, UK) and X-ray photoelectron spectroscopy (XPS, Thermo K-Alpha XPS, Thermo Fisher Scientific, Waltham, MA, USA) of Yeungnam University Center for Research Facilities. The XRD patterns were recorded using Cu-Kα radiation (*λ* = 0.154178 nm) between the 2*θ* of 20° and 70° (step size: 0.02°). XPS was performed using a monochromatic Al-Kα X-ray source (*hυ* = 1468.6 eV). The chamber pressure was maintained at approximately 10^−8^ mbar, and the spectra were obtained from a spot size of 400 μm with the energy steps of 0.1 eV. The electrical characteristics of the TFTs were measured using a Keithley 4200 SCS (Cleveland, OH, USA) at room temperature in the vacuum state (approximately 10^−3^ torr) with the dark state to exclude the effects of H_2_O, O_2_, and visible light on the TFTs.

## 3. Results and Discussion

### 3.1. Patterning of EHD Jet-Printed ZTO Layers

**Figure 1:**
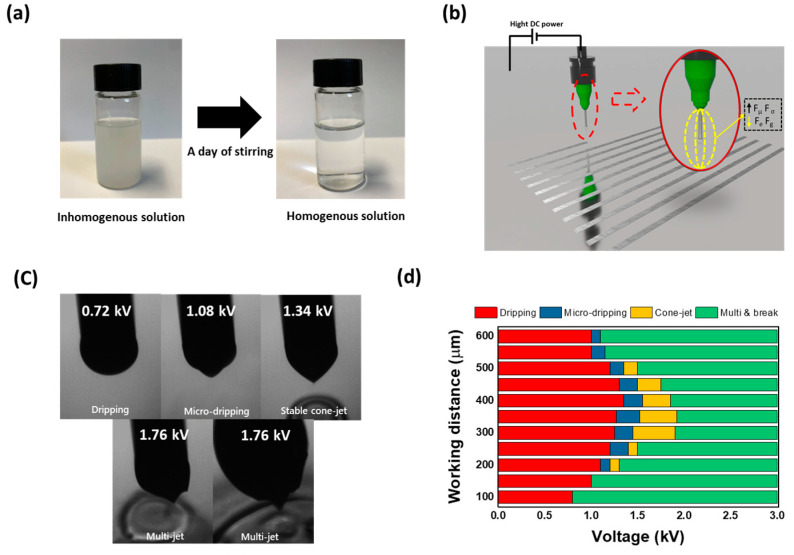
(**a**) ZTO ink for the printing process with as-fabricated and stirred one, and (**b**) schematic illustration of EHD jet printing process. (**c**) Various jetting behaviors, depending on the electrical bias and fabricated lines with micro-dripping and cone-jet mode. (**d**) Summary plots, including the EHD jet printing modes of ZTO dependent on the applied voltage and operation distance.

Before proceeding with ZTO printing in earnest, we must prepare the precursor solution. The precursor solution was synthesized using totally 0.4 M of zinc acetate dehydrate and tin chloride at the molar ratio of 1:1 in 2-methoxyethanol. A mixture of recently mixed substances was fairly turbulent; however, a day of stirring resulted in a homogeneous solution, depicted in Figure 1a. Using the thus synthesized solution, direct patterning was performed via the EHD jet printing process. The EHD jet printing technology utilizes the electrospinning behavior obtained at the short nozzle-to-substrate distance of approximately hundreds of micrometers [23]. Electrospinning is a fiber-production method that uses electric force to draw the charged threads of polymer solutions or polymer melts up to the fibers with the diameters in the order of some hundred nanometers. Likewise, in EHD jet printing depicted in Figure 1b, a force balance was achieved among the viscous force *F_μ_*, surface tension *F*_σ_, electrical force *F*_e_, and gravitational force *F*_g_. [16,24] Therefore, increasing the applied *F*_e_ induced charge accumulation on the surface of the fluid, and increasing the *F*_e_ resulted in the formation of an ellipsoidal meniscus, meaning breaking the balance of *F*_e_ and *F*_g_ with *F*_μ_ and *F*_σ_ of loaded ink. Notably, if the applied electrostatic field increased to some extent, the micro-droplet hung on the nozzle tip decomposed with various jetting behaviors that depended on the printing parameters such as electrospinning phenomena. 

According to the aforementioned principle, the synthesized ZTO ink was loaded into the EHD printer to find the optimum condition for the printing process with the working distance of 300 μm and flowrate of 0.15 μL/m. As depicted in Figure 1c, various jetting behaviors were achieved by applying different electric-field conditions. When the applied voltage was less than 0.72 kV, the dripping mode arose because the charges induced because of this low electrical force did not affect droplet formation but resulted only in a bulging drop that hung at the edge of the nozzle tip by the gravitational force. Subsequently, increasing voltage (1.08 kV) resulted in a micro-dripping jetting behavior in which the diameter of the droplet was considerably smaller than that of the capillary, and made a circular shape of printing on the substrate (Figure S1). When *F*_e_ + *F*_g_ was greater than the *F*_σ_ + *F*_μ_ of ZTO solution and the viscosity of the solution was sufficient to sustain the electrical pulling forces, a conical shape (Taylor cone) was observed in the stable cone-jet mode, thereby inducing the continuously uniform line printing, as depicted in Figure S1 [25,26]. Further increasing the voltage resulted in the formation of multi-jet modes in which the ZTO jet contained a broad distribution of drops and disintegrated because of the unstable force balance [27]. Therefore, we might fabricate various jetting behaviors of the synthesized ZTO ink by controlling the applied voltage. Moreover, these phenomena were adjusted by the distance between substrate and nozzle-tip, which critically influenced the *F*_e_ in printing condition [28]. Thus, we further investigated the jetting behaviors of the ZTO ink according to both operation distance and applied voltages, as illustrated in Figure 1d. The printing condition was optimized with these further investigations, and printed films were fabricated with stable cone-jet mode with 1.34 kV in working distance and flow rate as 300 μm and 0.15 μL/m. 

**Figure 2:**
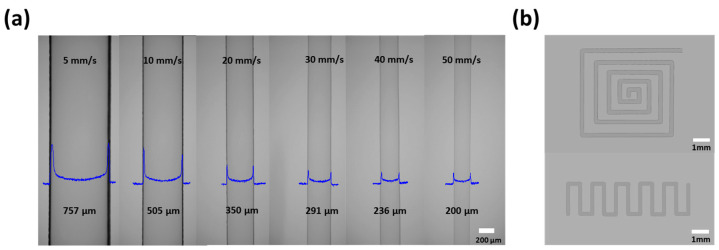
(**a**) Optical Microscope (OM) images of ZTO solution line patterning by the cone-jet mode of EHD jet printing method according to the printing speed (blue line shows the cross-sectional profile of line patterns). (**b**) Various shapes of patterns derived using the 50 mm/s condition of EHD jet printing.

The width of the patterns could be also controlled by varying the coating velocity [28]. Therefore, we varied the coating velocity of the ZTO solution from 5 to 50 mm/s. The optical microscopy (OM) images of the ZTO lines according to the coating speed are depicted in Figure 2a. Upon increasing the printing speed, the width of the ZTO line dramatically decreased: The line widths were 757, 505, 350, 291, 236, and 230 μm for the printing speeds of 5, 10, 20, 30, 40, and 50 mm/s, respectively. This decreasing trend in the line widths was attributed to the decrease in the volume of solution deposited per unit time, as the increase in the printing speed obstructed line spreading [29,30]. Besides, the printed ZTO patterns showed volcano shape due to the coffee-ring effect, but almost similar thickness (~100–120 nm scales) in the middle part of printing (see the cross-sectional profiles of each pattern in Figure 2a). Also, this direct patterning technology via EHD jet printing resulted in well-defined ZTO patterns with various shapes with no agglomerations (see Figure 2b), and such possibilities might expand the scope of applications of the EHD jet printing.

### 3.2. ZTO Active Layer Fabrication

**Figure 3:**
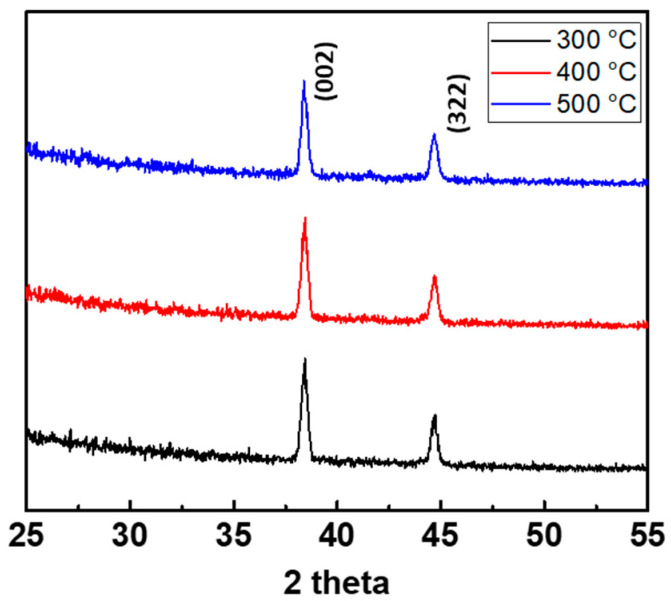
XRD profiles of the printed ZTO films at various annealing temperatures.

To utilize them as the active layers of metal-oxide TFTs, the precursors of ZTO were subjected to annealing to form semiconducting metal oxides at various temperatures (300 °C, 400 °C, and 500 °C) in ambient air condition for 1 h. The contents of the precursor were SnCl_2_ and Zn(CH_3_COO)_2_∙2H_2_O, and formed a metal oxide via a sol-gel reaction [31,32]. As mentioned previously in the experimental section, the solution used in this study was fabricated using the molar ratio of 50:50 of Zn and Sn oxide precursors. According to the previous reports, increasing the Sn contents in ZTO solution induced the fabrication of ZTO crystalline structure as (ZnO)_1−*x*_(SnO_2_)*_x_*, (Zn_2_SnO_4_)_1−*x*_ (ZnO)*_x_*, and (ZnSnO_3_) _1−*x*_(SnO_2_)*_x_*, among others [33]. As depicted in Figure 3, the 300 °C, 400 °C, and 500 °C cases of ZTO film exhibited the intense ZnO (002) peak at 38.4° and ZTO polycrystalline cubic (322) peak at 44.7°, which indicated the ZTO semiconductive crystals’ formations from the precursor of ZnO and SnO_2_ [34]. 

**Figure 4:**
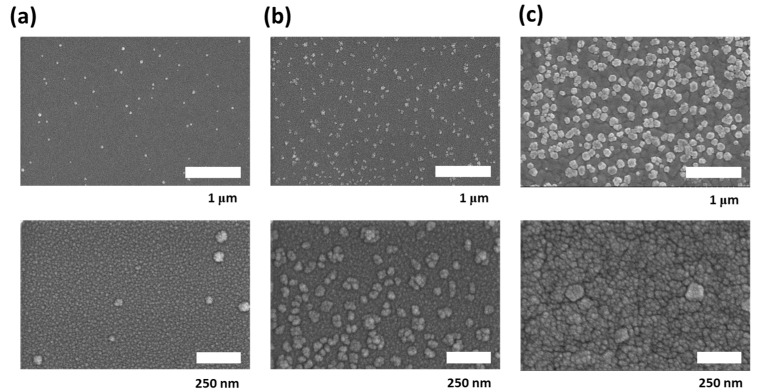
SEM images of the printed ZTO films according to the annealing temperature: (**a**) 300 °C, (**b**) 400 °C, and (**c**) 500 °C (upper: Overall image; lower: Magnified image).

To further understand the formation of the crystalline structure of our ZTO films, SEM and XPS measurements were performed. The morphological images of ZTO at various annealing temperatures are depicted in Figure 4. As shown in Figure 4a, the ZTO patterns with the annealing temperature of 300 °C induced ZTO crystallites of small and unclear particles with the size of less than 40 nm. However, upon increasing the annealing temperature, the growth or size of ZTO crystals dramatically increased (see Figure 4b,c). Especially when the temperature reached 500 °C, the size of the crystals became 180 nm. Therefore, although no change occurred in the crystal structure, a difference was observed in the crystal growth. 

**Figure 5:**
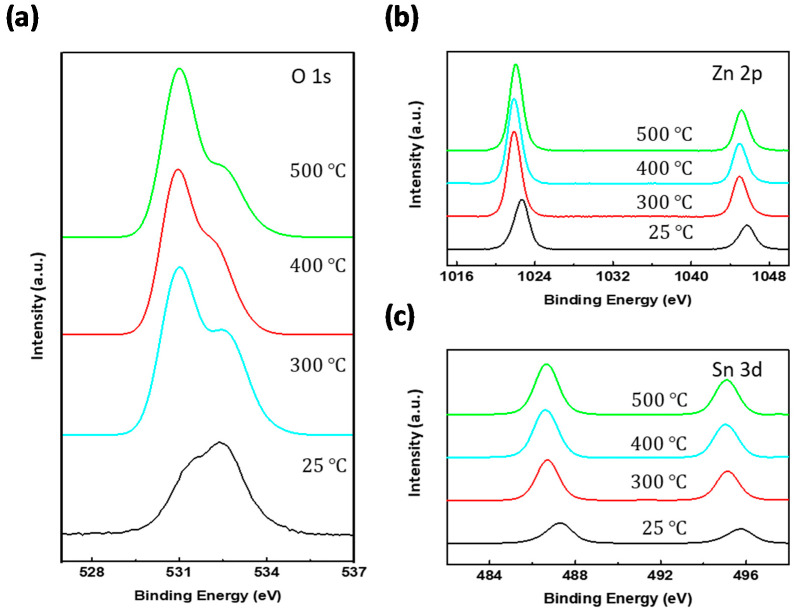
XPS spectra of the printed ZTO at various annealing temperatures: (**a**) O 1s, (**b**) Zn 2p, and (**c**) Sn 3d core levels.

In addition, the XPS data for various annealing conditions were carried out to study the chemical bonding of ZTO films. Particularly, the O 1s XPS data showed the tendency of the direct formation of ZTO oxide. As depicted in Figure 5a, the shape of the graph changed with the annealing temperature. The oxide peak related to the O^2−^ ions that were associated with neighboring zinc and tin atoms (*O_X_*) was located at 530 eV (blue vertical line) and gradually increased upon increasing the annealing temperature. Similarly, the peak generated due to oxygen vacancy (*O_VAC_*) or hydroxyl groups (*O_OH_*) was located at 531 eV with orange vertical line and gradually decreased [17]. Particularly, the annealed ZTO film showed a tendency for oxide peaks to be superior to oxygen vacancy or hydroxyl groups, where the as-deposited samples were considerably dominant. Furthermore, peak split analysis was conducted to confirm the effect of the annealing temperature on the formation of ZTO. 

**Figure 6:**
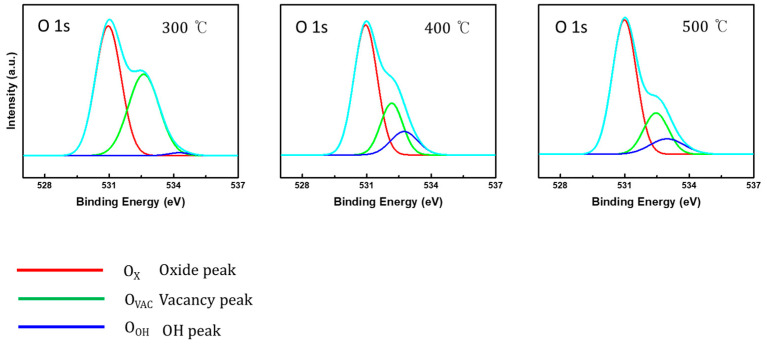
XPS spectra of the O 1s core shell of EHD jet-printed ZTO thin films showing peak distribution with various temperature: 300 °C, 400 °C, and 500 °C.

The O 1s peak in ZTO films was distributed to the following three types of chemical bonding: *O_X_*, *O_VAC_*, and *O_OH_*, as mentioned earlier. From this distribution, we could extract the metal-oxide ratio of ZTO by calculating the value of chemical-bonding type per (*O_X_* + *O_VAC_* + *O_OH_*). Using this relationship and the plots described in Figure 6, the oxide values of ZTO were calculated as 56.42%, 62.96%, and 67.69% for the annealing temperatures of 300 °C, 400 °C, and 500 °C, respectively. These values directly showed higher production of semiconducting ZTO upon increasing the thermal treatment temperature. In more detail, Table 1 shows the proportion of oxygen-involved bonds; the proportion outlines how ZTO was formed. At 300 °C, despite the smallest oxide ratio (56.42%) among the three values, the proportion by hydroxyl (1.06%) was smaller than those by others (13.33% and 11.01% for 400 °C and 500 °C, respectively). This result was attributed to the precursor properties that the formation of metal oxide for SnCl_2_ and Zn(CH_3_COO)_2_∙2H_2_O occurred at 320–430 °C and 35–300 °C, respectively [31]. Therefore, the formation of the metal oxide occurred in earnest at the temperature of 300 °C or more, and, consequently, hydroxyl groups were generated according to the sol-gel reaction and gradually decreased upon fabricating metal oxides. In addition, the Zn 2p_3/2_ (1021 eV) and Sn 3d_5/2_ (486 eV) peaks were increased because of the thermal treatments (see Figure 5b,c).

### 3.3. ZTO Active and MWCNT S/D-Based TFT

**Figure 7:**
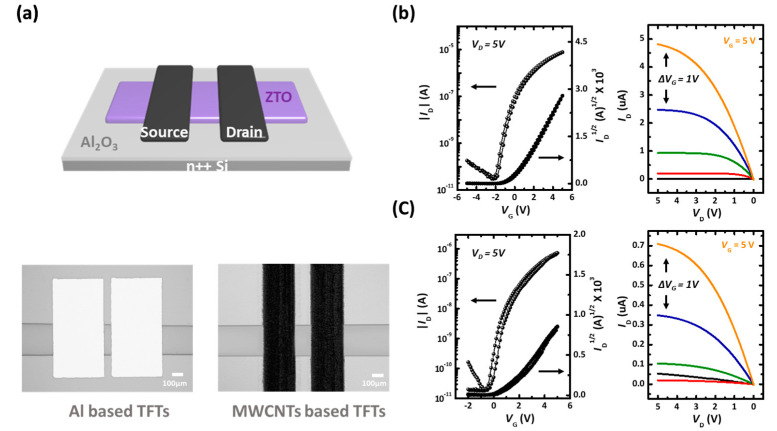
(**a**) Device configuration and OM images of EHD jet-printed ZTO. The transfer and output characteristics of ZTO devices with (**b**) Al and (**c**) MWCNTs S/D electrodes.

The electrical properties of the EHD jet-patterned ZTO were evaluated by fabricating typical bottom-gate top-contact TFTs using a typical metallic material (Al) and printable solution-based material (MWCNTs). ALD deposited Al_2_O_3_ 50 nm (capacitance *C_i_*: 96.6 nF/cm^2^) were utilized as the gate dielectric layers of TFTs, and 10 layers of ZTO patterns were patterned via the cone-jet mode of EHD jet printing process for active layers (30 mm/s, 300 μm, and 0.5 μL/min for printing velocity, working distance, and flow rate of ink). Then, the printed ZTO layers were annealed in various temperature conditions (300 °C, 400 °C, and 500 °C) for 1 h under ambient air conditions. The device fabrication was completed upon the deposition of S/D electrodes with Al and MWCNTs, as depicted in Figure 7a. The channel length (*L*) and width (*W*) defined using the effective length and width of ZTO patterns, respectively, existed between the S/D electrodes, as shown in Figure 7a (*W*/*L* were 4.0 for Al and 2.0 for MWCNTs, respectively). The devices were driven in the saturation regime with the gate voltage *V_G_* ranging from −5 or −2 to 5 V and S/D voltage *V_D_* of 5 V. The transfer electrical properties of the devices were extracted using the following relationship between drain current *I_D_* and *V_G_*: *I_D_* = *μ_FET_ C_i_ W*/2*L* (*V_G_*−*V_th_*)^2^, where *V_th_* denotes threshold voltage.

Previously analyzed part illustrated that 500 °C annealing process of ZTO is most suitable for fabrication of TFT devices. Indeed, the fabricated TFTs with 300 and 400 °C showed the insulating properties, as shown in Figure S2. Thus, the our applied sol-gel-based ZTO layers could be complete thermal decomposition under 500 °C annealing condition, and can be applied as a semiconductor layer in this case. In the case of 500 °C, thermally annealed ZTO TFT with conventional vacuum deposited Al electrode displayed the typical n-type transistor operation properties that show electrical performance with the *μ_FET_* of 1.35 ± 0.14 cm^2^ V^−1^ s^−1^, ON/OFF ratio of 10^5^, *V_th_* of −0.42 ± 0.37 V, and low operation voltage below 5 V (see Figure 7b). Especially, the operating devices exhibited the hysteresis behavior level with Δ*V_th_* of 0.46 V, meaning the difference between the forward and reverse swept transfer curves. In addition, the output results of ZTO TFTs were fully saturated and well matched with the saturation current value of the transfer curve, indicating reliable TFT operation.

Furthermore, we utilized the patterning of ZTO active layers with printable electrodes to fabricate TFTs. Following the previously proposed methods [35], we could fabricate the conductive layers as an S/D electrode, as depicted in Figure S3. Using this technique, it is possible to manufacture TFTs with semiconductor and conductive layer patterns through a printing process (see the right OM images in Figure 7a). In Figure 7c, we depicted the transfer and output characteristics of a specific sample of each of the ZTO TFTs with MWCNTs S/D electrodes. Similar to Al electrode devices, the TFT showed the typical operation behavior of electron-based transistors with low operation characteristics. However, the electrical performance was inferior to that of Al-based devices, which yielded the *μ_FET_* of 0.52 ± 0.08 cm^2^ V^−1^ s^−1^, ON/OFF ratio of 10^4^, and *V_th_* of 0.70 ± 0.25 V. This is because the work function of MWCNTs-based electrode (approximately 4.95 eV) was higher than that of the conventional Al electrode (approximately 4.0–4.28 eV) [19,36,37]. Therefore, the higher work-function value of the electrode hindered the injection of electrons, which were the major charge carriers in our ZTO devices, from the electrode to semiconducting layers, thereby degrading the electrical performance of the devices. Despite the output curve being almost saturated, it showed fairly reliable characteristics, such as showing a saturation-current value that matched the saturation-current value shown in the transfer curve (see Figure 7c). 

**Figure 8:**
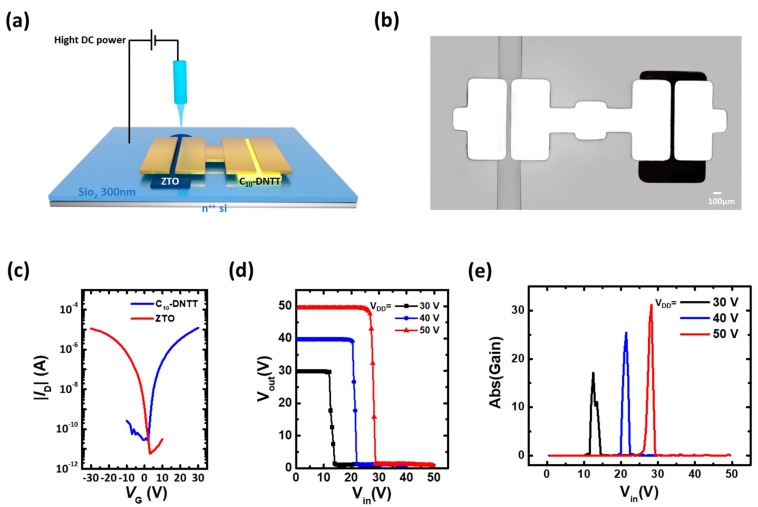
(**a**) Schematic showing and (**b**) OM image of complementary inverter with printed ZTO (left side) and C_10_-DNTT-based transistors (right side). The *W* and *L* for both transistors were 2000 μm and 100 μm, respectively. (**c**) Transfer characteristics (*V_D_* = ± 30 V) of ZTO and C_10_-DNTT transistors. (**d**) Voltage transfer characteristics and (**e**) DC voltage gains of complementary inverters with various *V_DD_* values.

Furthermore, we fabricated the complementary inverter with conventional p-type organic field effect transistors to identify the benefits of EHD-printed ZTO transistors for manufacturing the integrated circuitry devices. The fabrication of complementary inverter was initiated with printing of ZTO active layers with previously optimized conditions. Then, the sequential deposition processes of p-type active layers (C_10_-DNTT) and gold electrodes through the vacuum deposition process were conducted to complete the fabrication of complementary inverter, as described in Figure 8a,b (schematics and OM image). Figure 8c revealed that the C_10_-DNTT p- and ZTO n-type transistors exhibited similar performances in respect of turn-on voltage and saturation currents (see the transfer characteristics of Figure 8c). Due to these balanced electrical characteristics of p- and n-type devices, their complementary inverters exhibited good voltage transfer characteristics under various *V_DD_*, as shown in Figure 8d. The inverters were operated with a sharp voltage inversion over various ranges of *V_IN_*, which was caused by the on and off of p- and n-type transistors. Besides, the voltage gains of inverter, defined as d*V_OUT_*/d*V_IN_*, were calculated from the operation plot in Figure 8c (see the graph in Figure 8d). The maximum gain value reached 31.2 with a *V_DD_* of 50 V, which was well operated, like the previously reported printed ZTO transistor-based inverter results [38,39].

## 4. Conclusions

We demonstrated the EHD jet printing technology-based direct patterning of ZTO semiconducting layers, as well as their usage as the active layers of TFTs with conventional and printable electrode materials. Highly homogeneous ZTO precursor ink was observed to be appropriate for the EHD jet printing process, yielding various jetting modes (dripping, micro-dripping, cone-jet, and multi-jet). In addition, the stable cone-jet mode induced various ZTO line patterns depending on the printing speed. Various analysis tools such as XRD, SEM, and XPS revealed the fabrication mechanism of our patterned ZTO films. In optimized conditions, EHD jet-printed ZTO lines with micro-scale dimensions were used to fabricate the conventional metal oxide-based TFTs, which showed satisfactory and reliable electrical performances with low hysteresis. Furthermore, we patterned MWCNTs’ lines onto pre-patterned ZTO samples via EHD jet printing and utilized them as the S/D electrodes of the TFTs. These EHD-printed TFTs, which comprised ZTO active layers and MWCNTs S/D electrodes, displayed reliable TFT driving properties. In particular, these optimized devices were successfully applied to the complementary inverter with p-type organic semiconductor-based transistors, showing good voltage transfer characteristics. 

## Figures and Tables

**Table 1:** Deconvoluted O 1s core shell of EDH jet-printed ZTO thin film in various temperature conditions.

	300 °C	400 °C	500 °C
*O_X_*/*O_X_* + *O_VAC_* + *O_OH_*	56.42%	62.96%	67.69%
*O_VAC_*/*O_X_* + *O_VAC_* + *O_OH_*	42.51%	23.71%	21.30%
*O_OH_*/*O_X_* + *O_VAC_* + *O_OH_*	1.06%	13.33%	11.01%

## Supplementary Materials

The following are available online at https://www.mdpi.com/2079-4991/10/7/1304/s1, Figure S1: OM images of ZTO-printed layers with micro-dripping and cone-jet mode. Figure S2: Transfer characteristics with ZTO TFTs with 300 and 400 °C thermal annealing condition. Figure S3: OM and SEM images of EHD jet-patterned MWCNTs.
