# Supplementary material for: Direct Patterned Zinc-Tin-Oxide for Solution-Processed Thin-Film Transistors and Complementary Inverter through Electrohydrodynamic Jet Printing
Source: Nanomaterials (Basel). 2020 Jul 3;10(7):1304. doi: 10.3390/nano10071304 (PMC7407936; doi:10.3390/nano10071304)
Supplement: Supplementary file 1 [file nanomaterials-10-01304-s001.pdf]

nanomaterials

Supplementary Material

Direct Patterned Zinc-Tin-Oxide for Solution-Processed Thin-Film Transistors and Complementary Inverter Through Electrohydrodynamic Jet Printing

Heqing Ye [1,†], Hyeok-Jin Kwon [2,†], Xiaowu Tang [1], Dong Yun Lee [3,*], Sooji Nam [4,5,*] and Se Hyun Kim [1,*]

[1] School of Chemical Engineering, Yeungnam University, Gyeongsan 38541, Korea; yeheqing5420@gmail.com (H.Y.); tangxiaowu@naver.com (X.T.)

[2] Department of Chemical Engineering, Pohang University of Science and Technology, Pohang 37673, Korea; hj1370@postech.ac.kr (H.-J.K),

[3] Department of Polymer Science and Engineering, Kyungpook National University, Daegu 41566, Korea

[4] Flexible Electronics Research Section, Electronics and Telecommunications Research Institute, Daejeon 34129, Korea

[5] Advanced Device Technology, University of Science & Technology, Electronics and Telecommunications Research Institute, Daejeon 34129, Korea

* Correspondence: dongyunlee@knu.ac.kr (D.Y.L.), sjnam15@etri.re.kr (S.N.), shkim97@yu.ac.kr (S.H.K.); Tel.: +82-5-3810-2788 (S.H.K.)

† These authors contributed equally to this work

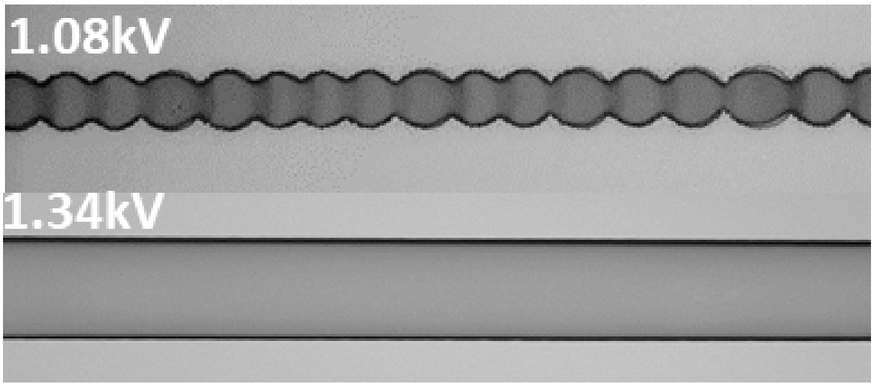

Figure S1. OM images of ZTO printed layers with micro-dripping and cone-jet mode.

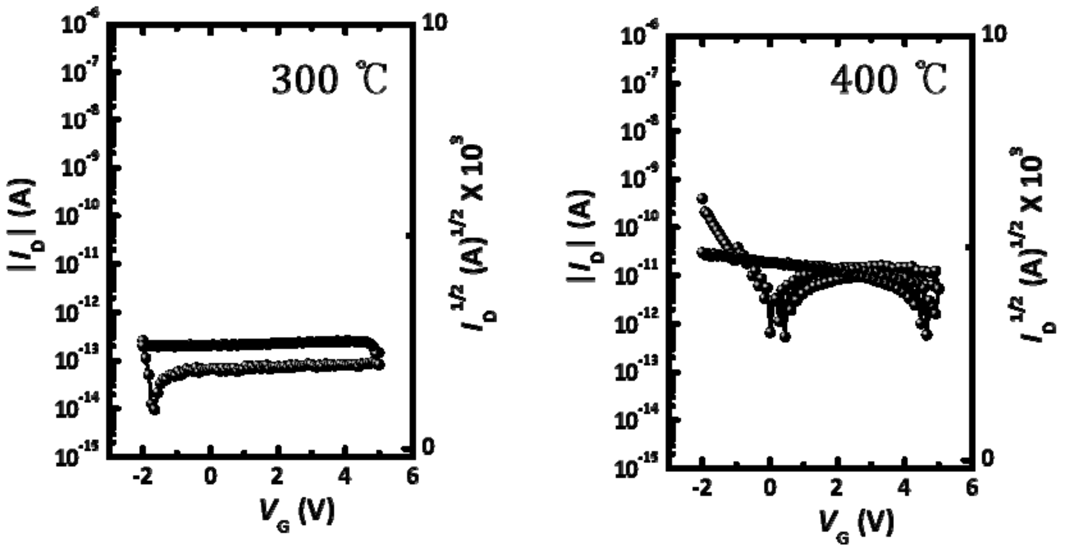

Figure S2. Transfer characteristics with ZTO TFTs with 300 and 400 °C thermal annealing condition.

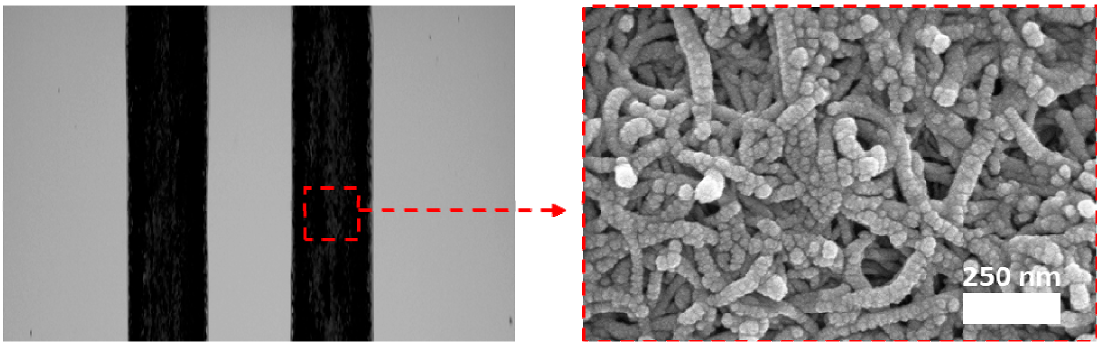

(a) (b)

Figure S3. (**a**) OM and (**b**) SEM images of EHD-jet patterned MWCNTs.

© 2020 by the authors. Licensee MDPI, Basel, Switzerland. This article is an open access article distributed under the terms and conditions of the Creative Commons Attribution (CC BY) license (http://creativecommons.org/licenses/by/4.0/).
